# Immunological Function of Sphingosine 1-Phosphate in the Intestine

**DOI:** 10.3390/nu4030154

**Published:** 2012-03-06

**Authors:** Jun Kunisawa, Hiroshi Kiyono

**Affiliations:** 1 Division of Mucosal Immunology, Department of Microbiology and Immunology, Institute of Medical Science, The University of Tokyo, Tokyo 108-8639, Japan; 2 Department of Medical Genome Science, Graduate School of Frontier Science, The University of Tokyo, Chiba 277-8562, Japan; 3 Graduate School of Medicine, The University of Tokyo, Tokyo 113-0033, Japan; 4 Core Research for Evolutional Science and Technology (CREST), Japan Science and Technology Agency, Tokyo 102-0076, Japan

**Keywords:** intestinal immunity, lipid, IgA antibody, intraepithelial T lymphocytes, food allergy

## Abstract

It has been shown that dietary materials are involved in immune regulation in the intestine. Lipids mediate immune regulation through a complex metabolic network that produces many kinds of lipid mediators. Sphingosine-1-phosphate (S1P) is a lipid mediator that controls cell trafficking and activation. In this review, we focus on the immunological functions of S1P in the regulation of intestinal immune responses such as immunoglobulin A production and unique T cell trafficking, and its role in the development of intestinal immune diseases such as food allergies and intestinal inflammation, and also discuss the relationship between dietary materials and S1P metabolism.

## 1. Introduction

It is generally accepted that dietary components are involved in immune regulation. The intestinal immune system, especially, seems to be directly affected by the digestion and absorption of dietary materials. Intestinal tissues are primary sites for infection by many pathogenic microorganisms, and commensal bacteria are abundant. Thus, the intestinal immune system has to create harmonious immunological condition, and the disruption of the intestinal immune homeostasis leads to the development of allergic, inflammatory, and infectious diseases [1,2].

Dietary lipids seem to be the dietary materials most involved in the regulation of intestinal immune responses after the conversion into lipid mediators [3]. Among various lipid mediators, sphingosine-1-phosphate (S1P) is a biologically active sphingolipid that regulates cell trafficking and activation [4,5]. S1P is abundantly present in the blood and lymph, which is originated from the cell membranes from sphingomyelin and is produced mainly by platelets, erythrocytes, and endothelial cells [6]. It is degraded by S1P lyase in the lymphoid tissues [7]. This metabolic pathway establishes an S1P gradient between the blood/lymph and lymphoid tissues and mediates cell trafficking.

The S1P gradient is recognized by cells expressing S1P receptors, and these cells migrate toward high concentrations of S1P. Of the five types of S1P receptor, type-1 S1P receptors (S1P1) are preferentially expressed by lymphocytes, and they determine lymphocyte emigration from and retention in the lymphoid tissues [8]. S1P1 is highly expressed in naive lymphocytes, including single-positive thymocytes expressing either CD4 or CD8, and expression is decreased upon lymphocyte activation. S1P1 expression recovers once the activated lymphocytes are fully differentiated and this recovery leads to their emigration from the lymphoid tissues into the blood circulation [4,5]. Studies indicate that the trafficking of macrophages, dendritic cells, and natural killer cells is mediated by S1P2, S1P3, and S1P5, respectively [9,10,11].

Recent studies have revealed additional functions of S1P in immune regulation that are independent of cell trafficking [4]. For example, differentiation of T cells is regulated by S1P1-mediated signaling [12,13,14]. It has also been demonstrated that a S1P2-mediated pathway is involved in the activation of mast cells [15] and macrophages [16], and that S1P3 are involved in dendritic cell endocytosis [10]. These findings together suggested that the S1P plays critical role in the activation and differentiation of immunocompetent cells involved in the both innate and acquired phases of immune responses in addition to their function of cell trafficking.

These biological and immunological functions show that S1P is involved in the maintenance of immunosurveillance as well as the development of immune diseases. In this review, we discuss the relationship between dietary materials (e.g., lipids, vitamin, and colorant) and S1P metabolism and describe the immunological functions of S1P, such as regulation of immunoglobulin A (IgA) production and intraepithelial T-lymphocyte trafficking, and its role in the development of intestinal immune diseases such as food allergy and intestinal inflammation.

## 2. Relationship Between S1P and Dietary Lipids

**Figure 1:**
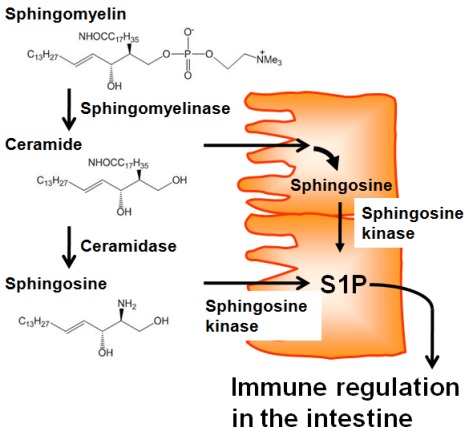
Dietary sphingolipids in epithelial-cell S1P production. Dietary sphingomyelin is degraded into ceramide and subsequently sphingosine by alkaline sphingomyelinase and ceramidase, respectively, which are expressed on the apical membranes of epithelial cells. In the epithelial cells, absorbed ceramide is metabolized into sphingosine. Together with absorbed sphingosine, sphingosine kinase metabolizes sphingosine into S1P, which then participates in immune regulation in the intestine.

Several lines of evidence demonstrate that intestinal tissues contain higher levels of sphingolipids, including S1P, than other tissues [17]. There is no evidence of intestinal uptake of sphingolipids from the blood, and germfree rats have comparable levels of sphingolipids in the intestine to conventional specific pathogen-free (SPF) rats [18]. Therefore, it is plausible that a source of sphingolipids in the intestine could be daily consumed diet. Adult humans ingest around 0.3 to 0.4 g sphingolipids per day, especially sphingomyelin from meat, milk, egg, and fish [19]. Dietary sphingomyelin is not directly absorbed, but is first degraded into ceramide and sphingosine [20,21] by alkaline sphingomyelinase and ceramidase, respectively, which are expressed on the apical membranes of epithelial cells [22,23]. Because epithelial cells express several key enzymes (e.g., sphingosine kinase) in the production of S1P from ceramide and sphingosine [23,24], it is possible that epithelial cells obtain ceramide and sphingosine from dietary sphingomyelin to produce S1P (Figure 1), thereby regulating intestinal immune responses and the associated intestinal immune diseases. Consistent with this, several studies showed that the incidence and severity of intestinal inflammation was changed by the uptake of dietary sphingomyelin [25,26] and the enzymatic activity of sphingomyelinase [27] and sphingosine kinase [28]. In addition, it was reported that dietary cholesterol inhibits the intestinal absorption of sphingolipids [29], implicating that cholesterol-rich Western diets may affect the availability of S1P precursors and consequently interfere with S1P-mediated intestinal immunity.

## 3. Regulation of S1P Metabolism by Dietary Materials

In addition to dietary lipids, other dietary materials are also involved in the regulation of S1P metabolism. For instance, S1P lyase, a key enzyme to degrade S1P and thus keep optimal S1P low concentration, requires vitamin B6 as a co-factor [7]. Thus, administration of vitamin B6 antagonist impaired S1P lyase activity, which consequently led to the defect of lymphocyte trafficking caused by inappropriate S1P gradient [7]. Similar effect was noted in 2-acetyl-4-tetrahydroxybutylimidazole (THI), a component of caramel food colorant III used in food products. THI inhibits S1P lyase and thus, like treatment with vitamin B6 antagonist, prevents normal lymphocyte trafficking [7]. These findings led to the use of THI for the treatment of immune diseases [30,31,32].

## 4. S1P Regulates Innate and Acquired Phases of Intestinal IgA Responses

IgA is the most frequently observed antibody isotype in the intestinal compartments and provides the first line of defense against pathogenic microorganisms invading through mucosal tissues. Therefore, the induction of appropriate IgA responses is a logical strategy for the development of oral vaccines [33]. Since IgA antibody is one of the major arms of the mucosal immune system in the digestive tract, which covers a large surface area, the intestinal IgA is originated from several induction sites including Peyer’s patches (PPs), isolated lymphoid follicles, and the peritoneal cavity [34].

**Figure 2:**
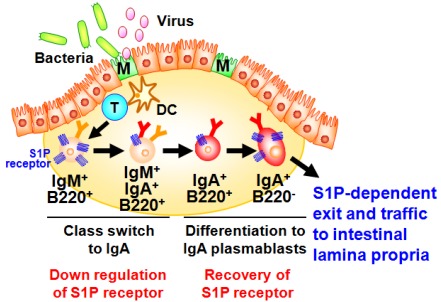
Sequential changes in S1P1 expression during B-cell differentiation in Peyer’s patches. Dendritic cells (DC) take the antigens transported by M cells from intestinal lumen and present them to T cells for their activation. Through the interaction with T cells and DCs, IgM^+^ naive B cells show class-switch from IgM to IgA. During this process, S1P1 is expressed at high levels in IgM^+^ naive B cells and downregulated on B cells class-switching from IgM to IgA and subsequently recovered on IgA^+^ B220^−^ plasmablasts, resulting in their emigration from the Peyer’s patches and traffic into the intestinal lamina propria.

A well characterized gut-associated lymphoid tissue (GALT) is PPs. PPs act as induction sites for the initiation of IgA responses against T-cell-dependent antigens [35]. PPs are covered with a specialized epithelium known as follicle associated epithelium (FAE) containing antigen-sampling M cells, which are responsible for the uptake and transport of antigens from the intestinal lumen to antigen-presenting cells such as dendritic cells (DCs) (Figure 2) [36]. Then, DCs capture antigens from the M cells, process and present them to T cells. It has been shown that the formation of PP DC-T cell clusters provide both cellular and molecular environment for the generation of IgA committed B cells in PPs [34]. In this pathway, some of the activated T cells differentiate into follicular helper T cells to help the antibody class switching of B cells in the germinal centers [34]. Because of the unique cytokine environment (e.g., TGF-β, IL-4, and IL-21) and continuous stimulation by commensal bacteria in the intestine, PPs have been shown to equip with efficient molecular and cellular environment for the spontaneous and continuous B cell class switching from IgM to IgA [34,35]. After class switching to IgA, B cells further differentiate into IgA plasmablasts and then migrate out from the PPs for their subsequent trafficking to the intestinal lamina propria, where they terminally differentiate into plasma cells producing dimeric (or polymeric) forms of IgA. This process mainly contributes to the development of T cell-dependent antigen-specific immune responses. Thus, the PP-mediated induction pathway is considered to be a major arm of the acquired IgA response [34].

Our investigation provided new evidence that S1P regulated the B cell trafficking in the PPs for the intestinal IgA production [37]. We initially found that S1P1 expression in B cells changes during differentiation in the PPs (Figure 2) [37]. High levels of S1P1 expression were detected in IgM^+^ naive B cells, and expression was down-regulated when B cells started class switching to IgA. The low expressions of S1P1 allowed newly class-switched IgA^+^ B cells to retain in the PPs for the sufficient differentiation into the IgA^+^ plasmablasts. S1P1 expression was restored on the IgA^+^ plasmablasts, resulting in their emigration from the PPs. Mice treated with FTY720, an immunosuppressant inducing S1P1 downregulation [38], show selective accumulation of IgA^+^ plasmablasts in the PPs, leading to the disturbance of continuous delivery of IgA committed B cells from the PPs to the lamina propria of intestine. Consequently, the decrease of same population in the intestinal lamina propria was noted, which associated with the reduction of intestinal antigen-specific IgA responses against orally immunized protein antigen [37].

In the IgA production pathway in the gut, peritoneal B cells are an additional source of intestinal IgA [39]. A number of peritoneal B cells belong to a unique B-cell subset, termed as B1 cells, which produces antibodies against T-cell-independent antigens such as lipids and polysaccharides. Because these T-cell-independent antigens are conserved in various microorganisms, B1-cell-derived antibodies undiscriminatingly react to commensal and pathogenic bacteria and prevent their attachment and invasion into the host. This reaction is opposite to antibody responses against protein antigen mediated by PP B cells, which show rigid specificity against microorganisms. Therefore, it has been considered that B1-cell-derived IgA is categorized as to be innate-type antibodies that recognize a wide range of microorganisms in the intestine [39]. 

Trafficking of peritoneal B1 cells into the intestine requires S1P-mediated signaling [40]. Like B cells in the PPs, peritoneal B1 cells identically expressed S1P1. Thus, trafficking of peritoneal B cells into the intestine and consequent production of intestinal IgA are diminished by treatment with FTY720, mainly because of the inhibition of B1 cell emigration from the parathymic lymph nodes, which drain to the peritoneal cavity [40]. This impaired trafficking in FTY720-treated mice was associated with the decreased IgA responses against phosphorylcholine (a T-cell-independent antigen) induced by oral immunization with heat-killed *Streptococcal pneumoniae* [40].

We also found that S1P-mediated regulation of peritoneal B-cell trafficking requires crosstalk with stromal cells in the peritoneal cavity [41]. This interaction mediated by adhesion molecules (e.g., ICAM-1 and VCAM-1) on stromal cells and the expression is regulated by NFκB-inducing kinase (NIK). Therefore, NIK-mutant aly/aly mice show decreased sensitivity to FTY720 in the regulation of peritoneal B-cell trafficking due to the impaired expression of adhesion molecules although peritoneal B1 cells in aly/aly mice expressed comparable levels of S1P1.

## 5. Distinct S1P Dependency of Trafficking of Intraepithelial T-Lymphocytes in the Gut

**Figure 3:**
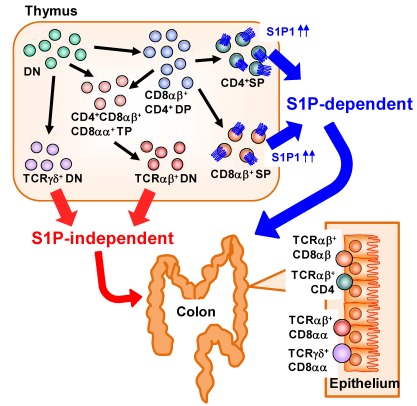
Distinct dependency on S1P in T-cell trafficking into the colonic epithelium. In the thymus, CD4^−^ CD8^−^ double-negative (DN) thymocytes differentiate into CD4^+^ CD8^+^ double-positive (DP) thymocytes and then into single-positive (SP) thymocytes expressing either CD4 or CD8 and αβTCR. These SP thymocytes express high levels of S1P1 and migrate out from the thymus and into the colon in an S1P-dependent manner. DN thymocytes express TCRαβ or TCRγδ. DN thymocytes expressing TCRαβ are derived from CD4^+^ CD8αα^+^ CD8αβ^+^ triple-positive (TP) thymocytes differentiated from DN or DP thymocytes. Little or no S1P1 expression is noted in the DN thymocytes expressing TCRαβ or TCRγδ, so traffic to the colonic epithelium proceeds in an S1P-independent manner.

Large numbers of lymphocytes are also present in the intestinal epithelium and called as intraepithelial lymphocytes (IELs) [42]. IELs are mostly T cells, but unlike in conventional T cells observed in the systemic compartments (e.g., spleen) which predominantly express the αβ T-cell receptor (αβTCR), in the IEL subset there is an abundance of T cells expressing the γδ T cell receptor (γδTCR) in addition to αβTCR^+^ T cells [42]. αβTCR recognizes peptide antigen presented via major histocompatibility complex (MHC) molecules, whereas γδTCR recognizes non-classical MHC molecules such as MHC class I chain-related proteins (MIC) A and B (MICA/B) in human and Rae-1 in mouse [43]. Unlike MHC molecules that act as ligand by presenting peptide antigen, non-classical MHC molecules act as a ligand by itself and the expression was induced by stress (e.g., infection, tumors, or chemical treatment) [44]. Thus, it is considered that αβTCR is involved in acquired immunity through the activation by specific presentation of antigenic peptides, whereas γδTCR is involved in innate immunity by the ligation of non-classical MHC molecules [42]. A distinctive pattern of CD8 expression has also been noted in IELs. Conventional αβTCR^+^ T cells express CD8 as a heterodimer of α and β (CD8αβ). In contrast, some IELs uniquely express CD8 as a homodimer (CD8αα) [42]. A previous study identified a unique precursor of CD8αα IELs in the thymus [45]. In the thymus, CD4^−^ CD8^−^ double-negative thymocytes differentiate into CD4^+^ CD8^+^ double-positive thymocytes and then further differentiate into single-positive thymocytes expressing either CD4 or CD8. CD8αβ^+^ IELs are derived mainly from CD8^+^ single-positive thymocytes expressing αβTCR. CD8αα^+^ IELs, however, originate from double-negative thymocytes expressing either αβTCR or γδTCR that have themselves differentiated from unique CD4^+^ CD8αα^+^ CD8αβ^+^ triple-positive thymocytes (Figure 3) [45].

S1P has been involved in the regulation of cell trafficking of different subsets of IELs originated from thymus. We found that each type of IEL shows a different dependency on S1P in its trafficking from the thymus to the intestine, especially in the colon (Figure 3) [46]. When mice were treated with FTY720, decreased numbers of CD8αβ^+^ IELs were observed. In contrast, the numbers of CD8αα^+^ IELs were barely affected. These data suggest that, in the colonic epithelium, CD8αβ^+^ IELs are S1P dependent and CD8αα^+^ IELs are S1P independent. Consistent with this finding, CD8^+^ single-positive thymocytes—the precursors of CD8αβ^+^ IELs—express high levels of S1P1 [8], whereas no S1P1 expression has been noted on double-negative thymocytes, the precursors of CD8αα^+^ IELs [46]. These findings suggest that S1P1 expression was different in different subsets of thymic precursors of IELs and provide versatile immunological pathways in the intestine.

## 6. S1P-Mediated Regulation in the Development of Intestinal Immune Diseases

**Figure 4:**
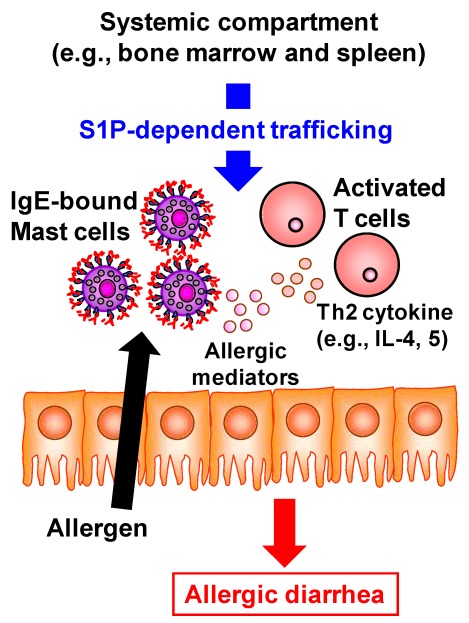
S1P mediates intestinal allergy by regulating pathogenic T and mast cell infiltration into the colon. In murine food allergy model, systemically sensitized T cells migrate into the colon upon the oral challenge with same allergen. This trafficking is mediated by S1P and thus treatment with FTY720 resulted in the inhibition of activated T cell trafficking into the colon. In the colon, these activated T cells produced high amounts of Th2 cytokines such as IL-4 and IL-5 for promotion of mast cell recruitment and proliferation. In addition, mast cell itself expresses S1P1. Therefore, FTY720 treatment directly and indirectly (Th2 cytokine from activated T cells) decreases the numbers of mast cells in the colon. These effects lead to the inhibition of allergic diarrhea.

Accumulating evidence has revealed the pivotal role of S1P in the development of inflammatory diseases such as autoimmune type 1 diabetes, rheumatoid arthritis, and multiple sclerosis [5]. FTY720 prevents the egress of autoreactive lymphocytes from the lymph nodes into the peripheral circulation and subsequent across the blood–brain barrier into the central nerve system and thus has recently been approved as an oral therapy for multiple sclerosis [47]. In addition to being involved in these immune diseases at the systemic immune compartments, S1P is involved in the development of intestinal immune diseases including food allergies and intestinal inflammation [5]. The number of patients with food allergies has increased not only in children but also in adults; the development of effective preventive and therapeutic strategies for food allergies is therefore required to improve patients’ quality of life. Using the ovalbumin-induced murine food-allergy model developed by our group [48], we examined the molecular and cellular mechanisms underlying the development of food allergies and found that, in allergic mice, activated T cells migrate into the colon, where they produced high amounts of Th2 cytokines such as IL-4 and IL-5 [48]. We demonstrated that the trafficking of pathogenic T cells from the systemic compartments into the colon was mediated by S1P (Figure 4) [49]. Indeed, activated T cells in the colon of allergic mice expressed S1P1 and their infiltration into the colon and subsequent production of Th2 cytokines (e.g., IL-4 and IL-5) were inhibited by the treatment with FTY720 [49]. In addition, the infiltration of mast cells, effector cells in the development of food allergy, into the colon was also prevented in the FYT720-treated mice [49]. As a mechanism of FTY720-mediated inhibition of mast cell infiltration, it was likely that FTY720 directly and indirectly prevented the mast cell infiltration into the colon. Direct effect of FTY720 was predicted by results that mast cells expressed S1P1 and their *in vitro* migration was inhibited by FTY720 [49]. Indirect effect is mediated by activated T cells producing Th2 cytokines which enhanced the proliferation and recruitment of mast cells [50]. Thus, inhibition of activated T cell trafficking into the colon by FTY720 resulted in the reduced recruitment and/or proliferation of mast cells. Taken together, involvement of S1P in the trafficking of both pathogenic T cells and mast cells is a potential target for prevention and treatment of food allergies.

Similarly, several lines of evidence have demonstrated that the FTY720 treatment prevents the development of intestinal inflammation [51,52,53]. For example, in a spontaneous colitis model in interleukin-10-deficient mice, administration of FTY720 suppressed the infiltration of pathogenic T cells producing interferon-γ [51]. Infiltration of the colon by pathogenic T cells was also inhibited by treatment with FTY720 in both a dextran sulfate sodium (DSS)-induced colitis model and a T-cell transfer model in mice [52,53]. Although S1P regulates the activation of several inflammatory cells via modulation of the signaling of certain innate receptors such as toll-like receptors, TNF receptor, and protease-activated receptor 1, and S1P itself is produced by activated inflammatory cells [4], collectively these findings suggest that S1P–S1P1 axis participates mainly in the development of intestinal immune diseases at the stage of pathogenic cell trafficking into the colon.

## 7. Conclusion

It is clear from past and current studies that S1P plays an important role in the regulation of the immune system of the gut in both healthy and disease states. In general, S1P is derived from sphingomyelin and is produced mainly by platelets, erythrocytes, and endothelial cells in the body. However, in the intestine, it is likely that epithelial cells contribute most to the production of S1P. Most importantly, S1P produced by epithelial cells seems to originate from dietary sphingolipids, especially sphingomyelin. Thus, elucidation of the complex networks established by dietary lipids will create a new era in nutrition-based mucosal immunology and should provide a new strategy against intestinal immune diseases.
